# Road infrastructure impacts on transportation safety (case study: Yazd rural roads)

**Published:** 2019-08

**Authors:** Abolfazl Khishdari

**Affiliations:** ^*a*^University of Yazd, Yazd, Iran.

## Abstract

**Background:**

Statistics showed that road traffic fatalities is amongst the top ten cause of. Road infrastructure quality play a vital role in reducing traffic related accidents. There are different ways to inspect road infrastructures. In Iran however road safety inspections are mostly based on periodic field surveys. This study goaled to investigate the importance of road infrastructure quality on transportation yearly crash rate index based on 3 years (2015-2017) police reported crash data with the case study of Yazd province. Infrastructure quality data are collected from Yazd department of transportation and road maintenance for Yazd rural highways. The recorded qualities were based on 4 point scale road deficit (i.e. very low, low, medium and high). The influence of road infrastructure quality were examined based on both descriptive statistics and crash rate index modeling. It was tried to compare ten different modeling structure based on akaike information criterion (AIC) and Bayesian information criterion (BIC). The models encompasses: Poisson, negative binomial (NB2), zero-truncated-poisson (ZTP), zero-truncated-negative-binomial (ZTNB), zero-inflated-poisson (ZIP), zero-inflated-negative-binomial (ZINB), REPoisson, RENB2, FEPoisson and FENB. Regardless of the yearly recorded road infrastructure data, investigations demonstrated that nearly 75% of road infrastructure qualities are in medium condition for all years.

**Results:**

Results showed that the FENB model outperform the competing models. Results also showed the reduction in road traffic crash rate index when road infrastructure quality decreased by one level form very low to low and also from low to medium. But there seems that the index will increase when road infrastructure quality decreased one level from medium to high.

**Conclusions:**

The whole results claims that we are in critical condition for our road infrastructure maintenance budget assignment.

**Keywords:**

Road infrastructure quality, Crash rate index, Crash modeling

